# Impairments of Motor-Cortex Responses to Unilateral and Bilateral Direct Current Stimulation in Schizophrenia

**DOI:** 10.3389/fpsyt.2013.00121

**Published:** 2013-10-04

**Authors:** Alkomiet Hasan, Theresa Bergener, Michael A. Nitsche, Wolfgang Strube, Tilmann Bunse, Peter Falkai, Thomas Wobrock

**Affiliations:** ^1^Department of Psychiatry and Psychotherapy, Ludwig-Maximilians-University Munich, Munich, Germany; ^2^Department of Psychiatry and Psychotherapy, University of Goettingen, Goettingen, Germany; ^3^Clinic of Clinical Neurophysiology, University of Goettingen, Goettingen, Germany; ^4^Centre of Mental Health, County Hospital Darmstadt-Dieburg, Groß-Umstadt, Germany

**Keywords:** schizophrenia, plasticity, tDCS, connectivity, LTP, LTD

## Abstract

Transcranial direct current stimulation (tDCS) is a non-invasive stimulation technique that can be applied to modulate cortical activity through induction of cortical plasticity. Since various neuropsychiatric disorders are characterized by fluctuations in cortical activity levels (e.g., schizophrenia), tDCS is increasingly investigated as a treatment tool. Several studies have shown that the induction of cortical plasticity following classical, unilateral tDCS is reduced or impaired in the stimulated and non-stimulated primary motor cortices (M1) of patients with schizophrenia. Moreover, an alternative, bilateral tDCS setup has recently been shown to modulate cortical plasticity in both hemispheres in healthy subjects, highlighting another potential treatment approach. Here we present the first study comparing the efficacy of unilateral tDCS (cathode left M1, anode right supraorbital) with simultaneous bilateral tDCS (cathode left M1, anode right M1) in patients with schizophrenia. tDCS-induced cortical plasticity was monitored by investigating motor-evoked potentials induced by single-pulse transcranial magnetic stimulation applied to both hemispheres. Healthy subjects showed a reduction of left M1 excitability following unilateral tDCS on the stimulated left hemisphere and an increase in right M1 excitability following bilateral tDCS. In schizophrenia, no plasticity was induced following both stimulation paradigms. The pattern of these results indicates a complex interplay between plasticity and connectivity that is impaired in patients with schizophrenia. Further studies are needed to clarify the biological underpinnings and clinical impact of these findings.

## Introduction

Despite more than 50 years of research, the availability of a diverse range of antipsychotics from different chemical classes, and the development of evidence-based treatment guidelines, a significant proportion of patients with schizophrenia suffer from treatment resistance (1). As a result, the application of non-invasive brain stimulation techniques as add-on treatment options for schizophrenia has gained much attention in the last decade. For instance, repetitive transcranial magnetic stimulation (rTMS) has been used for the treatment of persistent auditory hallucinations and negative symptoms. However, as discussed elsewhere this results have been inconsistent (2) and meta-analyses have reported moderate to low effect sizes for these interventions (3). More recently, transcranial direct current stimulation (tDCS) has been introduced to treatment studies in patients with schizophrenia (4–10). Compared to rTMS, tDCS offers the possibility of non-focal brain stimulation with a current flow between two electrodes (anode and cathode) and allows the active stimulation of two locations on the scalp (11). tDCS modulates spontaneous neuronal activity via a subthreshold tonic depolarization (anodal tDCS) or hyperpolarization (cathodal tDCS) of neuronal membranes (11–14). Both animal and human studies have indicated that these polarity changes are related to the molecular processes underlying long-term potentiation (LTP) and long-term depression (LTD) (15–20). The classical electrode montage placed one electrode over the primary motor cortex (M1) and the other electrode over the contralateral orbitofrontal cortex (OFC) (11, 15). This M1-OFC setup has been established as the gold standard for motor-cortical stimulation, with alternative setups (e.g., left M1 – right M1) shown to be ineffective in early studies for short stimulation durations (11, 15). However, recent publications have shown that bilateral application of tDCS on the primary motor cortices can in fact be effective in modulating cortical excitability on both hemispheres in healthy humans (21). Imaging studies indicate that unilateral and bilateral tDCS have fundamental differences in their physiological mode of action. Bilateral tDCS applied to M1 in both hemispheres has been found to cause a decrease in interhemispheric functional connectivity during the stimulation session, but an increase in intracortical functional connectivity beyond the stimulation period (22). In contrast, unilateral stimulation results in the same online-effects, but induces no changes that persist following the termination of stimulation (22). Furthermore, as with other transcranial brain stimulation techniques, tDCS only modulates cortical excitability at the stimulated site, but also impacts interconnected cortical areas (e.g., via transcallosal fibers) (23, 24). The possibility to modulate the excitability balance between two hemispheres, or more specifically between two cortical areas, for treatment purposes has gained considerable attention. Indeed, it has been specifically investigated in the context of neuropsychiatric disorders such as stroke (bilateral stimulation of both M1) (25, 26), depression (bilateral stimulation of both DLPFCs) (27, 28), and schizophrenia (left DLPFC, left temporo-parietal cortex) (5). In schizophrenia, treatment studies or case reports [e.g., Ref. (5, 6, 9)] have mainly used a unilateral tDCS montage, although some case reports using a bilateral tDCS montage (e.g., left tempo-parietal cortex, right supraorbital area, or left and right prefrontal cortex) have also been reported (4, 7).

Previous physiological tDCS studies showed that classical unilateral cathodal tDCS failed to induce LTD-like plasticity on both the stimulated and non-stimulated hemispheres, indicating a deficient response capacity of the motor cortex and impairments in functional connectivity in patients with schizophrenia (29, 30). Furthermore, LTP-like responses following anodal tDCS were found to be abolished in chronically ill individuals but not in recent-onset patients with schizophrenia, and these deficits were associated with altered inhibitory networks (31). Studies using other stimulation techniques are compatible with the tDCS findings, with patients with schizophrenia displaying reduced motor-cortical plasticity following paired-associative stimulation (32) and inhibitory 1 Hz-rTMS (33), as well as diminished use-dependent plasticity (34).

Given the observed impairment in cortical plasticity and the theories of an association between impaired plasticity and dysconnectivity in patients with schizophrenia (35, 36), bilateral stimulation may offer an interesting opportunity to impact positively on these disturbed networks. However, little is known about the physiological underpinnings of bilateral tDCS in patients with schizophrenia. Therefore, the aim of the present proof-of-concept study was to determine the difference in the efficacy of bilateral and unilateral tDCS applied to the primary motor cortices in patients with schizophrenia compared to healthy controls.

## Materials and Methods

### Subjects

Ten patients with paranoid schizophrenia and 10 age- and sex-matched healthy subjects participated in this study after giving informed consent. As a recent publication reported that differences in handedness modulate the effects of tDCS and hemispheric connectivity (37), the left-handed subjects (one in each group) were excluded from analyses, leading to a total sample size of 18. Subjects with a history of dermatological diseases, dementia, neurological illnesses, severe brain injuries, or brain tumors were excluded from the study. The local ethics committee approved the protocol in accordance with the Declaration of Helsinki.

The clinical consensus diagnosis of schizophrenia was made independently by two psychiatrists of the study group (Alkomiet Hasan and Thomas Wobrock), based on the ICD-10 criteria. All subjects underwent a standardized test of hand preference (38) and patients also had their psychopathology (Positive and Negative Syndrome Scale, PANSS) (39), disease severity (Clinical Global Impression, CGI) (40), and psychosocial functioning (Global Assessment of Functioning, GAF) (41) assessed.

All patients with schizophrenia were receiving treatment with second-generation antipsychotics [seven in monotherapy, two combination treatment (one first-generation antipsychotic)]. No other concomitant CNS-active medications, other than biperiden were allowed. Chlorpromazine (CPZ) equivalents were calculated for each patient (42).

### tDCS procedure

Transcranial direct current stimulation was applied, using a commercially available DC stimulator (Eldith-Electro-Diagnostic and Therapeutic Systems GmbH, Ilmenau, Germany), through saline soaked surface sponge electrodes (35 cm^2^). For unilateral tDCS, the cathode was placed over left M1 and the anode was located contralaterally above the right orbit. Bilateral tDCS was performed by placing the cathode over left M1 and the anode over right M1. A continuous current flow of tDCS, with an intensity of 1 mA, was applied for 9 min in the unilateral and for 13 min in the bilateral configuration. For unilateral tDCS, the application of 9–13 min was shown to produce long-lasting reduction of M1 excitability (up to 1 h) (14, 16). The decision for 9 min unilateral tDCS was made to allow the comparison with other tDCS studies in schizophrenia [e.g., Ref. (29, 30)] and the decision for 13 min bilateral tDCS was made to be in the time frame for long-lasting anodal after-effects (14).

### TMS procedure and monitoring of excitability changes

To determine M1 excitability with single-pulse motor-evoked potential (MEP) measurements before and after tDCS, a previously described setup was used and adopted for the bihemispheric measurements (29, 30). Subjects were seated in a comfortable reclining chair with a passive arm support. Electromyographic (EMG) recordings from the right and left first-dorsal interosseous (FDI) muscles were made with surface electrodes. Raw signals were amplified, bandpass-filtered (2 Hz–10 kHz), and digitized using a commercially available amplifier. Each EMG recording was manually analyzed offline. TMS was performed with a biphasic MagPro X 100 magnetic stimulator (Medtronic Co., Copenhagen, Denmark) and TMS was applied to the hand area of the left and right motor cortices with a standard figure-of-eight magnetic coil. In accordance with other studies, the coil was held tangentially to head with the handle pointing backward and at an angle of 45° lateral to the midline. This setup induced a posterior-anterior directed current in the cortex.

The optimal coil position was defined as the stimulation site that produced the largest MEP at moderately suprathreshold stimulation intensities in the resting FDI muscles. The optimal positions were marked to ensure that the coil was held in the correct position and orientation throughout all experiments.

Single-pulse TMS before and after each stimulation procedure was performed at an intensity that evoked MEPs of about 1 mV (S1 mV, peak to peak, 0.7–1.3 mV) over left and right M1. We measured 30 MEPs at baseline, and 1 and 15 min after stimulation. Baseline cortical excitability was further determined only before stimulation by measuring the resting-motor threshold, short-latency intracortical inhibition (SICI), and cortical silent period (CSP). SICI was recorded with a standardized paired-pulse protocol (conditioning stimulus: 80% RMT, test stimulus: S1 mV, interstimulus interval (ISI): 3 ms) (43). We performed 10 trials with 3 ms ISI and the 30 MEPs were used as test pulse comparison. The effect of the conditioning stimulus on MEP amplitude of the test stimulus was determined by calculating the ratio of the average amplitude of the conditioned paired-pulse MEP to the average amplitude of the unconditioned single-pulse test MEP. Finally, CSP was measured in the tonically active FDIs (25–30% of maximal contraction) by stimulating the contralateral motor cortex with an intensity of 120% RMT. Ten trials were performed and the mean CSP duration calculated.

### Experimental design

**Figure 1:**
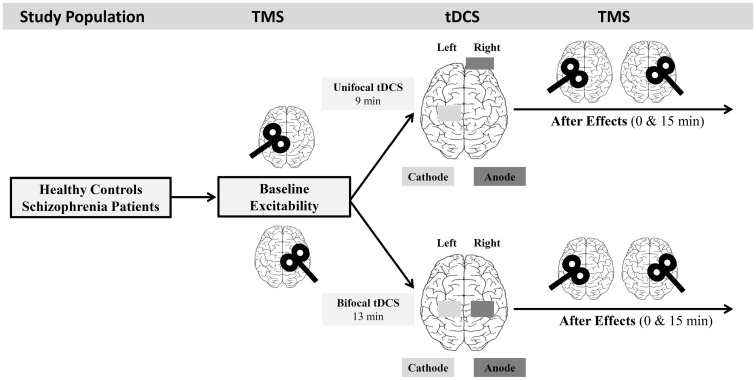
**Experimental course and study design**. All subjects participated in two experimental sessions (unilateral DCS, bilateral tDCS). Corticospinal excitability was assessed before and after tDCS stimulation on both hemispheres.

To compare unilateral and bilateral tDCS in both study groups, all 18 subjects were tested on two different days (time-interval 3–7 days), resulting in 36 experimental sessions. The study was designed as a balanced, complete-crossover study in a repeated measurement design (Figure 1).

### Statistical analyses

SPSS 20 for Windows was used for all analyses, and the level of significance was defined as α = 0.05. For gender, Chi square tests were computed to test for group differences. Independent-samples *t*-tests were used to compare mean ages and baseline excitability (S1 mV, RMT, SICI, CSP) between the groups. A repeated-measures ANOVA (RM-ANOVA) was calculated with the between-subject variable “group” (healthy subjects vs. patients with schizophrenia) and the within-subject variables “timecourse” (baseline, post-stimulation 1 and 15 min), “hemispheres” (left, right), and “stimulation” (bilateral, unilateral). Since there was only a trend-level interaction in the overall ANOVA for “stimulation × timecourse × group” when we examined the data points post-tDCS, we averaged all time-points to give a mean post-tDCS excitability measure (“time”) for all consecutive statistical analyses. To determine more specifically whether the MEP amplitudes before and after tDCS differed within- and between-groups, hemispheres, and stimulation types, Student’s *t*-tests (independent-samples for the intergroup comparisons, and paired-samples for the intragroup pre versus post comparisons, two-tailed, *P* < 0.05) were performed where appropriate (significant interactions in the ANOVA). *T*-tests were not adjusted for multiple comparisons in cases of significant interaction in the RM-ANOVAs. Sphericity was tested using Mauchly’s test and, if necessary (Mauchly’s test <0.05), the Greenhouse–Geisser correction was applied. Pearson correlations between dependent variables and CPZ equivalents were performed in the patient group. Data are presented as mean ± SD unless otherwise indicated.

## Results

### Sociodemographic variables and clinical ratings

Analyses revealed no group differences for age (*p* = 0.455) and gender distribution (*p* = 1.000). Patients with schizophrenia were all stable (at least 1 week with the same medication; medications stable for both measures) and showed moderate positive and negative symptoms and moderate degree of illness and impairment. Demographic and clinical characteristics as well as CPZ equivalents of the study groups are presented in Table 1.

**Table 1:** **Demographic and clinical characteristics of the subjects**.

Variable	*n*	Healthy controls	Schizophrenia patients	Statistics
Subjects (*n*)	18	9	9	
Age (years)	18	28.9 ± 12.5	34.0 ± 10.2	*p* = 0.455^a^
Gender (f/m)	18	3/6	3/6	*p* = 1.000^b^
**PANSS score**
Total	9	–	58.1 ± 19.9	
Positive	9	–	12.1 ± 6.8	
Negative	9	–	16.6 ± 4.2	
General	9	–	29.4 ± 11.1	
GAF	9	–	57.6 ± 15.1	
CGI	9	–	4.1 ± 1.7	
CPZ (daily)	9	–	453.7 ± 284.5	
DUP (years)	9	–	6.4 ± 6.1	

n = Number of subjects; f = female; m = male; r = right; l = left; PANSS = Positive and Negative Syndrome Scale; GAF = global assessment of functioning; CGI = clinical global impression; CPZ = chlorpromazine equivalent dose; DUP = duration of psychosis; ^a^independent-samples t-test; ^b^Chi square test. Data are presented as mean ± SD.

### Baseline excitability for both hemispheres

Independent *t*-tests between both groups comparing the left and right hemispheres revealed no significant differences for S1 mV, RMT, MEP amplitudes, SICI, or CSP (see Table 2). However, patients with schizophrenia had a numeric reduction in SICI on both hemispheres and a numeric prolongation in CSP of the left hemisphere. Although this can be explained by the small sample size, it is in line with many previous studies [for review see Ref. (44)].

**Table 2:** **TMS baseline parameters**.

Variable	*n*	Healthy controls	Schizophrenia patients	Statistics
**LEFT HEMISPHERE**				
RMT (%)	18	46.8 ± 7.6	46.1 ± 18.8	*p* = 0.922
S1 mV (%)	18	54.6 ± 8.2	57.3 ± 23.4	*p* = 0.742
CSP (ms)	18	138.0 ± 23.9	112.8 ± 42.1	*p* = 0.137
SICI 3 ms (%)	18	23.7 ± 26.4	50.7 ± 26.4	*p* = 0.261
**RIGHT HEMISPHERE**				
RMT (%)	18	37.7 ± 16.1	42.3 ± 24.9	*p* = 0.791
S1 mV (%)	18	46.8 ± 18.8	50.7 ± 30.3	*p* = 0.748
CSP (ms)	18	126.0 ± 24.0	126.1 ± 38.5	*p* = 0.994
SICI 3 ms (%]	18	40.1 ± 57.0	55.1 ± 87.4	*p* = 0.681

Statistics = independent t-tests; RMT = resting-motor threshold; S1 mV = intensity to evoke a MEP of ∼1 mV; CSP = contralateral silent period; SICI = short-latency intracortical inhibition. Data are presented as mean ± SD.

### Effects of plasticity induction

**Figure 2:**
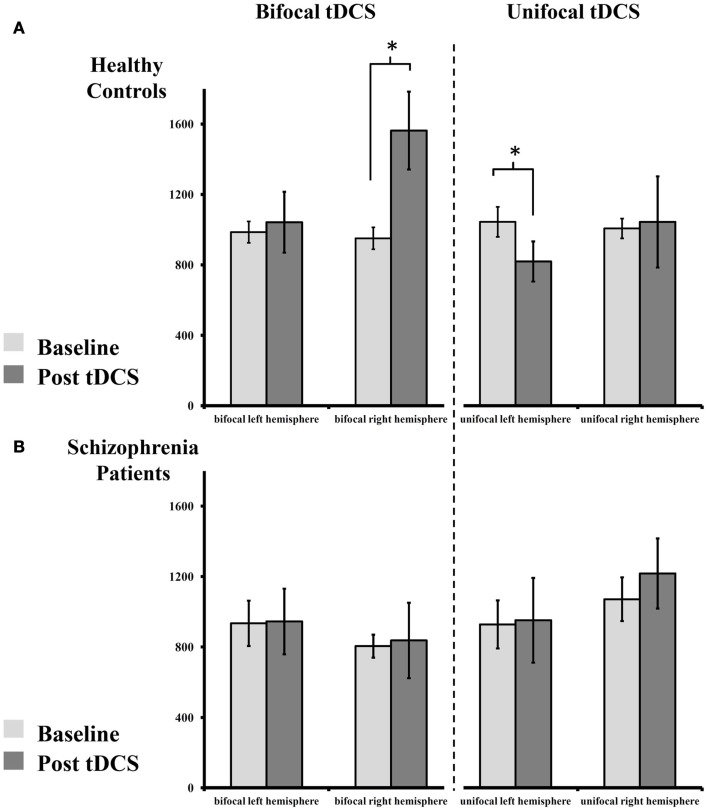
**Absolute change of MEP size pre- and post-tDCS**. **(A)** Healthy controls display an increase of MEPs after bilateral tDCS on the right (anodal), but not on the left hemisphere (cathodal). Following unilateral tDCS, healthy controls show reduced MEPs on the stimulated left, but not on the non-stimulated right hemisphere. **(B)** Patients with schizophrenia show abolished plasticity following both stimulation paradigms on both hemispheres. For details please see Section “Results.”

Since there was only a trend-level interaction “stimulation × timecourse × group” in the first RM-ANOVA, we pooled the post-tDCS MEP measures for a compound mean analysis (Figures 2A,B). The results of this ANOVA are presented below (Table 3). The RM-ANOVA with the main factors “stimulation,” “hemisphere,” “time,” and “group” revealed significant effects for the “stimulation × time × group” interaction (*p* = 0.027), trend-level interactions for “stimulation × hemisphere × group” (*p* = 0.083), “stimulation × time” (*p* = 0.090), and “hemisphere × time” (*p* = 0.081), but no further interactions or main effects (all *p* ≥ 0.119). Based on the “stimulation × time × group” interaction, further analyses were conducted. However, because no significant interactions involving the factor “hemisphere” were detected, no analyses between hemispheres were performed.

**Table 3:** **Results of the RM-ANOVAs for MEP values**.

	Hypothesis *df*, error *df*	*F* value	*p* Value
MEP-amplitudes (baseline, mean post)
Time	1, 16	1.327	0.266
Hemisphere	1, 16	2.157	0.161
Stimulation	1, 16	0.001	0.980
Time × group	1, 16	0.195	0.266
Hemisphere × group	1, 16	0.762	0.396
Stimulation × group	1, 16	2.710	0.119
Time × hemisphere	1, 16	3.475	0.081^#^
Time × stimulation	1, 16	3.254	0.090^#^
Hemisphere × stimulation	1, 16	0.464	0.506
Time × hemisphere × group	1, 16	1.710	0.209
Time × stimulation × group	1, 16	5.922	0.027*
Hemisphere × stimulation × group	1, 16	3.422	0.083^#^
Time × hemisphere × stimulation	1, 16	0.261	0.616
Time × hemisphere × stimulation × group	1, 16	1.102	0.309

The analyses show a significant “time × stimulation × group” interaction for MEP amplitudes and trend-level findings for “time × hemisphere” and “time × stimulation.” In cases of lacking interactions, no further *t*-tests were conducted. *p ≤ 0.05; ^#^*p* ≤ 0.09.

In the healthy control group, *paired-sample t-tests* showed an increase in MEP amplitude following bilateral tDCS on the right (anodal) [*t*_(8)_ = 2.710, *p* = 0.027], but not on the left (cathodal), hemisphere [*t*_(8)_ = 0.344, *p* = 0.740]. After unilateral tDCS, MEPs were reduced on the left hemisphere (cathodal) [*t*_(8)_ = 2.683, *p* = 0.028], but not on the non-stimulated right hemisphere [*t*_(8)_ = 0.136, *p* = 0.895]. Contrary to these findings, patients with schizophrenia showed no modulation of MEP amplitude following unilateral or bilateral tDCS on the left [all *t*_(8)_ < 0.132, all *p* > 0.899] and right hemispheres [all *t*_(8)_ < 1.235, all *p* > 0.252].

For bilateral tDCS, *independent-samples t-tests* showed no MEP baseline differences between healthy controls and patients with schizophrenia on the left [*t*_(16)_ = 0.364, *p* = 0.721] or right [*t*_(16)_ = 1.623, *p* = 0.124] hemispheres. After stimulation, no differences between groups could be detected on the left hemisphere (cathodal) [*t*_(16)_ = 0.382, *p* = 0.708]. However, on the right hemisphere (anodal), healthy controls showed significantly higher MEP values after stimulation compared to patients with schizophrenia [*t*_(16)_ = 2.359, *p* = 0.031].

For unilateral tDCS, *independent-samples t-tests* again showed no MEP baseline differences between healthy controls and patients with schizophrenia on the left [*t*_(16)_ = 0.722, *p* = 0.481] or right [*t*_(16)_ = 0.469, *p* = 0.645] hemispheres. Additionally, after tDCS no differences across groups could be detected on the left [*t*_(16)_ = 0.501, *p* = 0.623] and right hemisphere [*t*_(16)_ = 0.531, *p* = 0.602].

### Influence of clinical symptoms and antipsychotic medication

Pearson correlations between dependent variables and CPZs revealed a positive correlation between CPZ equivalents and MEP amplitudes at baseline (unilateral, right hemisphere) (*p* = 0.047). The detected correlations were, however, non-significant after correction for multiple analyses, and thus have to be interpreted with caution.

## Discussion

The results of this first proof-of-concept study comparing classic unilateral cathodal tDCS with simultaneous bilateral tDCS indicate that cortical plasticity is impaired in patients with schizophrenia following both stimulation protocols. Unilateral cathodal tDCS reduced MEP amplitudes on the stimulated hemisphere in healthy controls, but not in patients with schizophrenia. No effect on the non-stimulated hemisphere following left M1 cathodal tDCS could be observed in either group. The recently introduced bilateral tDCS paradigm (here: cathodal tDCS over the left M1 and anodal tDCS over the right M1) facilitated MEPs on the right hemisphere but had no effect on the left hemisphere in healthy controls. However, in patients with schizophrenia, no excitability modulation following tDCS could be observed on either hemisphere. The pattern of our results indicates global deficits of cortical plasticity in the schizophrenia brain and offers new insights into the interplay between plasticity and connectivity in this population.

### Bilateral tDCS for bihemispheric excitability modulation in healthy controls

The initial paper by Nitsche and Paulus (15) tested different electrode positions and found the maximum effect for anodal and cathodal stimulation placing the motor cortex stimulation electrode over the left M1 and the return electrode on the contralateral supraorbital side. However, they did not observe any excitability-modulating effects using bilateral motor cortex electrode montage during a short DC stimulation for 4 s, which does not induce after-effects (15). A recently published study was able to show an excitability-modulating effect of bilateral tDCS applied to both motor cortices (21). Mordillo-Mateos et al. (21) investigated systematically bilateral M1 tDCS and showed that bilateral tDCS enhanced cortical excitability on the anodal-stimulated side and decreased cortical excitability at the cathodal-stimulated site, irrespective of whether anode and cathode were placed over left or right M1. The bilateral setup in our study led to an excitability-enhancing effect of anodal tDCS applied to the right M1, but we were not able to show an excitability-diminishing effect following cathodal tDCS applied to left M1. The differences between both studies could possibly be explained by the stimulation duration (5 min compared to 13 min bilateral tDCS in our study) and the stimulation intensity (2 mA compared to 1 mA in our study). It is well-established that modulation of these parameters impact on the after-effects that follow unilateral tDCS (11, 15, 45) and it is very likely that related effects occur using the bilateral electrode montage. Another study reported a MEP increase following both unilateral and bilateral anodal tDCS applied with 1 mA for 13 min to the left M1, with no differences between protocols. However, right M1 excitability was not reported (46). Our results are in line with recent publications indicating a modulatory effect on M1 excitability following bilateral tDCS in healthy subjects, with methodological differences across studies accounting for diverse results. Further systematic evaluations are needed to clarify the biological impact of bilateral tDCS.

### Impaired cortical plasticity in patient with schizophrenia following both stimulation paradigms

Cathodal unilateral tDCS failed to reduce MEP amplitudes on the stimulated hemisphere in patients with schizophrenia. This has already been shown by our group in large samples (29, 30) and this present result confirms these findings. However, in the present study we were not able to show differences in MEP modulation between healthy controls and patients with schizophrenia on the non-stimulated hemisphere. In both groups, no effect on the non-stimulated right M1 could be observed following cathodal tDCS applied to left M1. This is in contrast to a study published by our group (30), but in line with an earlier study conducted in healthy subjects (47). Both our present study and that published by Lang et al. had rather small sample sizes (*N* = 9, respectively *N* = 8) and, therefore, these studies might not have enough statistical power to detect such subtle modulations which are subject to large between-subject variability. Bilateral tDCS enhanced MEPs on the anodal site (right M1), but had no effect on the cathodal site (left M1) in healthy subjects. However, in patients with schizophrenia, bilateral tDCS on neither the left nor the right hemisphere was able to modulate cortical excitability, indicating deficient cortical plasticity, at least at the anodal site. To the best of our knowledge, only one study has investigated the physiological effects of anodal tDCS on M1 excitability in patients with schizophrenia so far. In this study, anodal tDCS applied to left M1 failed to increase MEP amplitudes in chronically ill patients, but led to a sufficient MEP-facilitation in early-disease-stage patients and healthy controls (31).

Impaired plasticity is one of the best established neurobiological theories of schizophrenia. The term plasticity refers to the brain’s capacity to respond to external stimuli and it is associated with modulations of synaptic activity, micro- and macro-connectivity, and neural integrity (48–50). The molecular processes underlying plasticity are LTP and LTD, and their functioning is critically dependent on the activity of *N*-methyl-d-aspartate-receptors (NMDAR) (51). A dysfunction of NMDARs, which results in a consequent hyper- or hypoglutamatergic state, has been proposed to underlie some aspects of schizophrenia’s pathophysiology (52). This glutamate hypothesis of schizophrenia is based on the observation that healthy subjects who received NMDAR antagonists showed clinical features of schizophrenia, including positive, negative, or cognitive symptoms (44, 52). Since NMDARs are critically involved in the neuroplastic after-effects that follow anodal and cathodal tDCS (17, 18), one could hypothesize that the observed effects of impaired cortical plasticity are possibly in line with the theory of dysfunctional NMDAR-activity in schizophrenia. From a physiological perspective, one could further hypothesize that a dysregulation in calcium homeostasis is responsible for the bidirectional effects on of cortical plasticity following tDCS in our study. It has been suggested that different calcium levels determine the development of three different forms of plasticity: LTD, LTP, and one zone with no plasticity development (“no man’s land”) (53, 54). Based on this model and that described in our previous publications (31, 55), one could speculate that either a hyperglutamatergic state with an excessive calcium influx or a reduction in NMDAR-activity resulting in receptor hypofunction, may account for the observed plasticity deficit. Finally, it may not be that impairments in the presynaptic glutamate neurotransmission alone explain plasticity deficits in patients with schizophrenia, but rather that deficits in postsynaptic activity of NMDAR (e.g., cell-receptor interaction) may also play a role (56, 57). Although a possible molecular theory for the observed plasticity deficits, this explanation remains speculative as NMDA transmission has not been addressed directly in this study.

One remarkable finding of the presented study is that both stimulation protocols failed to induce plasticity in patients with schizophrenia. The complex interplay between plasticity and connectivity is at the core of the popular theory published by Stephan et al. (36). In their theory, impairments of NMDAR-activity lead to reduced synaptic plasticity, with consequent disturbances in micro- and macro-connectivity in the developing brain influencing all neurotransmitter systems and leading to aberrant discharge in neural systems (36, 58). This dysconnectivity hypothesis combines available evidence from structural and functional disconnection studies of schizophrenia. For example, a reduction in white matter in the frontal and temporal lobes has been shown consistently in MRI studies (59). These observations are supported by neuropathological studies in patients with schizophrenia that indicate a loss of myelin-producing oligodendrocytes (60) or a reduced density of dendritic spines (61) in the frontal lobe, both of which are findings that can be closely associated with impaired neural connectivity. Furthermore, as recently reviewed, a functional hyperconnectivity involving different cortical and subcortical pathways has been linked to auditory and verbal hallucinations in schizophrenia (62). Therefore, both hypo- and hyperconnectivity have been postulated as explanations for the pathophysiology and symptomatology underlying schizophrenia.

Our setup does only allow the indirect measurement of M1–M1 connectivity (23) and both, impaired M1 plasticity as well as a disrupted M1–M1 connectivity can explain the observed results. Furthermore, it is very likely that the excitability changes in both motor cortices interact following both stimulation setups and that the direction of the changes are subject to considerable variability dependent on many, as yet unidentified, factors (63). Therefore, the final interpretation of our preliminary results has to remain speculative. Further studies with a special attention on intra- and intercortical M1 physiology are needed to further clarify the association of cortical connectivity and plasticity following non-invasive brain stimulation in schizophrenia. Due to the small sample size, we were not able to show differences in cortical excitability (RMT, SICI, CSP) between groups, but the numeric differences indicate an inhibitory deficit in patients with schizophrenia, as has been reported previously (44). Reduced inhibitory function has been shown to affect cortical plasticity following cathodal and anodal tDCS (29, 31), and it could be speculated that a related mechanism may explain the presented results. To sum up, our findings offer a new view on NMDAR-dependent cortical plasticity and highlight the difficulty associated with inducing plasticity in the schizophrenic brain with a single session of non-invasive brain stimulation.

### Limitations

For a comprehensive discussion of our observations, some important limitations have to be taken into account. First, the impact of antipsychotic medication on cortical plasticity following tDCS must be highlighted as the most important possible confounding factor. This has been discussed elsewhere (31), but in brief, the reader should be aware that, in pharmacological studies modulating the dopaminergic system in healthy subjects, a single-administration of dopamine agonists/antagonists indicate that dopaminergic modulation has a strong impact on the after-effects of tDCS (64–67). All our patients received second-generation antipsychotics which influence the dopaminergic system. However, one should note that the cited studies used a single drug-administration in healthy subjects and it is clear that the underlying mechanisms in patients with schizophrenia receiving a long-term treatment are different. Nonetheless, further studies investigating M1 excitability following non-invasive brain stimulation have shown a plasticity deficit in both medicated and unmedicated patients with schizophrenia (33, 34). The second limitation relates to the proof-of-principle design of this study. Though being within the range of other studies in the field, our sample size is rather small, leading to some numeric differences between groups that do not reach significance (see Results; Table 3). Therefore, the results need to be confirmed in larger studies with better statistical power. Next, due to feasibility reasons, we focused principally on cathodal tDCS applied to the dominant hemisphere. Further studies need to investigate unilateral and bilateral anodal tDCS applied to left M1. The reason for our cathodal-setup was that schizophrenia is a disorder of impaired inhibitory function and that our foregoing studies have shown stable results using the unilateral setup (44). Furthermore, we decided to use different stimulation durations for unilateral and bilateral tDCS to allow comparability to other tDCS studies in schizophrenia (9 min unilateral cathodal tDCS) and to be within the time frame of long-lasting after effects of anodal tDCS (13 min of anodal tDCS). As both stimulation times are within the known optimal time frames for unilateral tDCS (14, 16), a confounding effect is not expected. However, a systematic evaluation of these parameters is needed and should be addressed in future studies. Finally, our bilateral setup presented here has not been previously evaluated using a sham condition in healthy subjects. Therefore, the lacking sham condition in our study needs to be considered as a limiting factor.

## Conclusion

This is the first study comparing the efficacy of unilateral and bilateral tDCS in patients with schizophrenia. In line with previous studies, it was not possible to induce cortical plasticity using the unilateral setup and the same was true for the bilateral setup. These results indicate complex deficits in plasticity and connectivity in patients with schizophrenia. It may be speculated that, due to intrinsic inhibitory dysfunction within the motor cortices, plasticity processes are impaired, leading to consecutive alterations in micro- and macro-connectivity. With regard to the possible clinical application of bilateral tDCS in schizophrenia, these results need to be taken into consideration. Assuming related deficits in brain areas other than M1, one could hypothesize that the efficacy of tDCS stimulation applied to the frontal lobe (e.g., negative symptoms, cognitive symptoms) or to the temporal lobe (hallucinations) might be reduced. It is tempting to try placing the anode on the DLPFC to enhance activity and the cathode on the temporal lobe to reduce activity, with a possible consequent network effect that improves positive and negative symptoms. Our results urge us to remain cautious and to pay more attention to the physiological underpinnings of tDCS in schizophrenia. However, the first clinical proof-of-principle trials and case reports using both unilateral (5, 9) and bilateral (4, 7) tDCS were promising, and it is likely that repeated tDCS will affect the schizophrenic brain in a different way to a single application. Further studies are needed to clarify the clinical significance of tDCS in schizophrenia and to identify likely responding and non-responding patients. In summary, our results indicate that prominent LTP- and LTD-plasticity deficits can be observed in patients with schizophrenia irrespective of the stimulation technique applied to elucidate them.

## Authors Contribution

Conception and design: Alkomiet Hasan, Michael A. Nitsche, and Thomas Wobrock. Performing experiments: Theresa Bergener Analysis of the data and interpretation of the results: Alkomiet Hasan, Theresa Bergener, Michael A. Nitsche, Thomas Wobrock, and Peter Falkai. Drafting of the article and literature search: Alkomiet Hasan, Theresa Bergener, Tilmann Bunse, and Wolfgang Strube. Final approval of the manuscript: all authors.

## Conflict of Interest Statement

The authors declare that the research was conducted in the absence of any commercial or financial relationships that could be construed as a potential conflict of interest.
